# 

**Published:** 1843-03

**Authors:** 


### CONTENTS

OF NO. II.

---

### ORIGINAL COMMUNICATIONS.

### ESSAYS AND CASES.

Art. I.—An Experimental and Critical Inquiry into the Nature and Treatment of wounds of the Intestines. By Samuel D. Gross, M. D., Professor of Surgery in the Louisville Medical Institute. - - - 161

### SELECTIONS FROM AMERICAN AND FOREIGN JOURNALS.

Engorgement of the Uterus. - - - - - 225
Muriate of Ammonia in Hemicrania. - - - - 227
Case of Idiopathic Hydrophobia. - - - - - 227
Operation for Enlarged Patellar Bursa. - - - - 229
Proto-sulphuret of Iron, a new Antidote for Corrosive Sublimate. - - - - - - - 230
Cure of Crooked Nose by Subcutaneous Division of the Cartilages. - - - - - - - 231
Mode of Treating Itch at Berlin. - - - - - 231

Sleep of Animals and Man. - - - - - 233
Case of Nasal Enlargement Successfully Treated. - 234

ORIGINAL INTELLIGENCE.

Travelling Editorials, - - - - - - 235

